# Prevalence of Ideal Cardiovascular Health Metrics among Young Asian Adults over 5 Years of Follow-Up

**DOI:** 10.3390/nu15030645

**Published:** 2023-01-27

**Authors:** Pu-Jun Fang, Ping-Hsuan Kuo, Wei-Liang Chen, Tung-Wei Kao, Li-Wei Wu, Hui-Fang Yang, Tao-Chun Peng

**Affiliations:** 1 Division of Family Medicine, Department of Family and Community Medicine, Tri-Service General Hospital, National Defense Medical Center, Taipei 11490, Taiwan; 2 Division of Internal Medicine, Tri-Service General Hospital, National Defense Medical Center, Taipei 11490, Taiwan; 3 Department of Internal Medicine, Taipei Veterans General Hospital, Taipei 11217, Taiwan; 4 Faculty of Medicine, National Yang Ming Chiao Tung University, Taipei 11221, Taiwan; 5 Division of Geriatric Medicine, Department of Family and Community Medicine, Tri-Service General Hospital, National Defense Medical Center, Taipei 11490, Taiwan; 6 Graduate Institute of Medical Sciences, National Defense Medical Center, Taipei 11490, Taiwan; 7 Graduate Institute of Clinical Medicine, College of Medicine, National Taiwan University, Taipei 11490, Taiwan

**Keywords:** ideal cardiovascular health, cardiovascular diseases, young adults, Asia

## Abstract

Background: Ideal cardiovascular health (CVH) metrics play an important role in preventing cardiovascular disease (CVD). However, there is a lack of cohort studies on CVH metrics among young Asian adults. The aims of this study were to describe early changes in CVH among young Asian adults and to investigate the association between CVH metrics and sociodemographic variables. Methods: A total of 10,000 young adults (aged 21–30 years) were recruited between 2000 and 2016. There were two CVH measurements taken from these participants over the study period. One measurement was taken at the beginning, and the other was taken five years later. Subgroup analysis of the changes in CVH metrics was divided by education level and marital status. Results: The mean age of the participants was 26.8 years. The initial prevalence of ideal CVH metrics was 52.3% and 86.8% and decreased to 43.8% and 81.2% after five years for males and females, respectively. In the subgroup analysis, males with less than a university education had a smaller ideal CVH metric decrease (6.2%) than males with more than a university education (8.9%), while females with more than a university education had a smaller ideal CVH metric decrease (5.4%) than females with less than a university education (7.3%). Married males had a smaller ideal CVH metric decrease (6.1%) than single males (9.1%), while single females had a smaller ideal CVH metric decrease (5.3%) than married females (6.2%). Conclusions: The prevalence of ideal CVH metrics among young adults gradually decreased as age increased. Higher educational attainment and unmarried status were associated with a greater prevalence of ideal CVH metrics regardless of sex, but early CVH changes differed by sex, education level, and marital status. The prevalence of CVH changes found early among young adults can be used to monitor CVH changes quickly. Effective health promotion programs are needed to maintain CVH metrics among young adults.

## 1. Introduction

The American Heart Association (AHA) defines ideal cardiovascular health (CVH) metrics based on seven behaviors and risk factors that reduce cardiovascular disease (CVD). These metrics are divided into lifestyle (i.e., body mass index (BMI), physical activity, diet, and smoking) and biological (i.e., blood glucose, blood lipids, and blood pressure) metrics [1]. The lower risk of cardiovascular events and all-cause mortality among adults is related to higher CVH metrics [2,3]. In addition to the incidence of CVD during adulthood, CVD incidence among young adults is also increasing. An analysis showed that CVH metrics were negatively associated with the incidence of CVD among young adults [4]. A large prospective cohort study also revealed that cardiovascular risk factors in childhood were associated with cardiovascular events in midlife [5]. Furthermore, there is an inverse relationship between carotid intima-media thickness (cIMT) and the number of CVH metrics among young adults, suggesting that CVH reflects young adults’ vascular health [6]. It is obvious that CVH metrics are equally important for adults of any age. The AHA proposed CVH metrics to monitor CVD risk among young adults and to reduce the burden of CVDs [7].

Several studies that have investigated the prevalence of CVH metrics and the influence of socioeconomic status on CVH among children and young adults showed that less than 1% of young adults achieved full scores in all seven CVH metrics [6,8]. A combined study that followed subjects aged 19–31 years since childhood demonstrated that higher family socioeconomic status in childhood predicted CVH in adulthood [8]. However, there are few young adult CVH studies to observe initial CVH changes; two exceptions are a Brazilian birth cohort and a Finnish study [9,10] but their participants’ ages were close to those of adolescents. We focused on young adults more than adolescents because young adults are in an essential life transition, including the completion of education, leaving the parental home, and entering the workforce [11]. These early crucial CVH changes may improve nonideal health behaviors or factors in advance. Leading an ideal lifestyle in early young adulthood is fundamental for healthy longevity, as behavior patterns become more difficult to change with age [12]. The prevalence of CVH changes found early among young adults can be used to monitor CVH changes quickly and remind young adults to pay attention to their living habits and health during young adulthood.

In addition, there are substantial cultural and socioeconomic differences between Western and Asian countries, which may have an impact on CVH [13]. Appropriate cultural understanding can benefit health promotion programs, so CVH metrics must be studied considering different cultures to unearth the most relevant factors for young adults [14]. To date, in Asian countries, there are only cross-sectional studies involving children and young adults [15,16]. There is no cohort study on CVH metrics among young adults in Asian countries. Additionally, this investigation aimed at examining how sociodemographic factors influence CVH. Health equality can highlight strategies targeting CVH differently according to sex, education level, and marital status. Therefore, the scope of this study was to study early changes in CVH metrics among young Asian adults and to investigate the association between CVH scores and sociodemographic factors.

## 2. Materials and Methods

### 2.1. Study Population and Study Design

The data for the present study were drawn from the MJ (měi jhào) Health Management Institution and were collected from 2000 to 2016. The MJ Health Management Institution, which has four branches (Taipei, Taoyuan, Taichung, and Kaohsiung) across Taiwan, is a private institute that provides periodic health examinations. All branches use the same screening protocols and test machines.

This study was based on a retrospective cohort design. The participants, aged 21 to 30 years, were enrolled in the analysis to evaluate the primary preventive effect of CVH metrics on young adults. There were two CVH measurements taken from these participants over the study period. One measurement was taken at the beginning, and the other was taken 5 years later. All the participants provided written informed consent for the use of their anonymized personal data for research purposes. This study was approved by the Institutional Review Board of the Tri-Service General Hospital.

### 2.2. Measurements in the MJ Database

Every participant in the MJ database needed to complete a questionnaire and medical screening. The questionnaire was composed of 100 questions on topics including family history, personal history, nutrition, lifestyle (smoking, drinking, exercise, sleep), and recent health conditions [17]. All of the data from the validated questionnaire were self-reported the same as other published studies [18,19,20]. Medical screening was performed by trained nurses and evaluated by doctors. The items included blood analysis, urine tests, body measurements (height, weight, BMI), and physical activity. The screening procedure was identical and performed on instruments of the same model. Blood samples were drawn with heparin as an anticoagulant, and serum samples were preserved at −20 °C. Overnight fasting blood was collected and analyzed (Hitachi 7150 autoanalyzers, Tokyo, Japan), with serum creatinine analyzed by the uncompensated Jaffe method with an alkaline picrate kinetic test [21]. Blood pressure was measured twice on the right arm with the subject in a sitting position after 5 min of rest using a computerized automatic mercury sphygmomanometer, Citizen CH-5000 (Citizen, Tokyo, Japan).

Leisure time PA was assessed using the validated MJ PA Questionnaire [22,23]. This questionnaire was similar to the International Physical Activity Questionnaire [24]. The reliability of our questionnaire is the same as that of other questionnaires that are widely accepted as reliable [25]. The questionnaire included exercise frequency, duration and intensity with several examples of activity types given for four intensity categories: light (e.g., slow walking), moderate (e.g., brisk walking), moderate-vigorous (e.g., jogging), or high-vigorous (e.g., running). We used the answers to assess the length of time spent performing physical activity in minutes each week. The details are provided in the references [17].

Food intake was collected by a self-report questionnaire created by MJ Health Management [17]. Diet was assessed through the certified and standard semiquantitative food frequency questionnaire (FFQ) [26]. Before data collection, the participants needed to answer 85 closed-ended questions about food consumption at different meal times during the month. According to the answers and hypothesized health effects, these were classified into 22 non-overlapping food groups the same as other studies [27,28,29]. The amount was assessed by the intake frequency data, and information on portion size was estimated with pictures of the measuring tools in each question. For example, the description of sugar-sweetened beverage consumption was “How many sugar-sweetened beverages do you drink, including coffee, tea, and Coke? (1 cup is equivalent to 240 mL)”. Each question had five choices for intake frequency, including “none or < 1 cup a week, 1–3 cups a week, 4–6 cups a week, 1 cup a day, or ≥ 2 cups a day”, from lowest to highest.

### 2.3. Cardiovascular Health (CVH) Metrics

CVH metrics included BMI, smoking status, healthy diet score, physical activity (PA), blood pressure, total cholesterol, and blood glucose based on a definition modified from the definition presented in the AHA guidelines [7]. We categorized the CVH metrics into ideal, intermediate, and poor. Ideal, intermediate, and poor smoking statuses were defined as never smoker, former smoker, and current smoker, respectively. PA in leisure time was calculated as min/week on the PA questionnaires. Ideal, intermediate, and poor PA was defined as ≥210 min/week, 60–210 min/week, and <60 min/week, respectively. BMI was calculated using measured weight (in kg) divided by height squared (in m^2^). Ideal, intermediate, and poor BMIs were defined as lower than 25 kg/m^2^, 25–29.99 kg/m^2^, and ≥30 kg/m^2^, respectively. The diet metric was composed of five items with the following components: (1) ≥300 g of vegetables and fresh fruits per day, (2) ≥200 g of fish per week, (3) ≥two 30-g equivalent servings of whole grains per day, (4) ≤1500 mg of sodium per day, and (5) ≥240 g of milk per day. Ideal, intermediate, and poor diet metrics were defined as 4–5 components, 2–3 components, and 0–1 components, respectively. Ideal, intermediate, and poor blood pressure (BP) levels were defined as systolic blood pressure (SBP) <120 and diastolic blood pressure (DBP) <80 mmHg, SBP 120–139 or DBP 80–89 mmHg, and SBP ≥140 or DBP ≥90 mmHg, respectively. Ideal, intermediate, and poor fasting serum glucose levels were defined as <100 mg/dl, 100–125 mg/dl, and ≥126 mg/dl, respectively. Ideal, intermediate, and poor total cholesterol levels were defined as <200 mg/dl, 200–239 mg/dl, and ≥240 mg/dl, respectively. Each CVH metric categorized as ideal received one point. The CVH score was calculated by the total number of ideal metrics, ranging from 0 to 7. Ideal cardiovascular health was defined as a score ≥4 according to previous studies, which showed that participants with <4 metrics had increased preclinical atherosclerosis [9,30]. Higher CVH scores indicated healthier participants.

### 2.4. Statistical Analysis

The characteristics of the participants were summarized as frequencies (%) for categorical variables and the means (standard deviations) for continuous variables. The characteristics of the study population and the changes in CVH metrics between sexes were compared using chi-square tests for categorical variables and t-tests for continuous variables. Subgroup analysis of the changes in CVH metrics was divided by education level and marital status. Moreover, we ran some multivariable logistic regression models of unmarried/married status and less than a university education/more than a university education. All of the statistical tests were two-tailed with *p* < 0.05. All statistical analyses were performed using the Statistical Package for the Social Sciences version 18.0 (SPSS, Inc., Chicago, IL, USA).

## 3. Results

The characteristics of the participants are summarized in Table 1. The mean age of the participants was 26.8 ± 2.4 years. Males had higher BMI, blood glucose, total cholesterol, blood pressure, and education levels. There were more married females than married males. The percentage of CVH metrics among females compared to males was higher in all categories except for ideal PA. Ideal diet and PA had the lowest proportions of participants.

Differences in CVH metrics after 5 years among the participants are presented in Table 2. After 5 years, the prevalence of CVH metrics shifted to nonideal, and scores clustered at approximately 3 to 4 for males and 4 to 5 for females. Among males, the largest reduction in CVH metrics was ideal total cholesterol, followed by ideal blood glucose, ideal BMI, and ideal PA. The largest increase in CVH metrics was observed for ideal BP followed by ideal diet. Among females, the largest reduction in CVH metrics was ideal total cholesterol and ideal blood glucose, followed by ideal BMI. The largest increase in CVH metrics was observed for the ideal diet followed by the ideal PA.

Initially, women’s diets were better than men’s diets. In the detailed ideal diet component (Supplementary Table S1), males had a more fiber-rich whole-grain diet than females, while women’s diets had fewer sodium- and sugar-sweetened beverages than men’s diets. A more ideal diet was observed for both sexes after 5 years.

After 5 years of follow-up, the prevalence of CVH metrics shifted to nonideal, but CVH metrics (score: 4, 6, 7) among females with a higher than university education unexpectedly increased (Table 3). Regarding individual CVH metrics, ideal BMI, glucose, and cholesterol significantly decreased among males with less than a university education. On the other hand, among males with more than a university education, ideal PA, BMI, glucose, and cholesterol significantly decreased, but ideal diet and BP significantly increased. Moreover, among females with less than a university education, ideal BMI, glucose, and cholesterol significantly decreased similar to those of their male counterparts, but ideal PA significantly increased. Ideal BMI, glucose, and cholesterol significantly decreased, but ideal PA and diet significantly increased among females with more than a university education.

After 5 years of follow-up, the CVH metrics shifted to nonideal, but CVH metrics (score: 4, 6, 7) in the single female group and CVH metrics (score: 6, 7) in the married male and female groups unexpectedly increased (Table 4). Regarding individual CVH metrics, the ideal PA in the single male group dropped sharply compared with that in the married male group. In contrast, the married female group’s ideal PA increased more than that of the single female group. The ideal diet in the single group increased sharply compared to that of either married group. Ideal BMI, blood glucose, and cholesterol all decreased regardless of sex and marital status. Ideal BP in the single male group increased after 5 years, while it decreased in the married male group but was nonsignificant.

**Figure 1:**
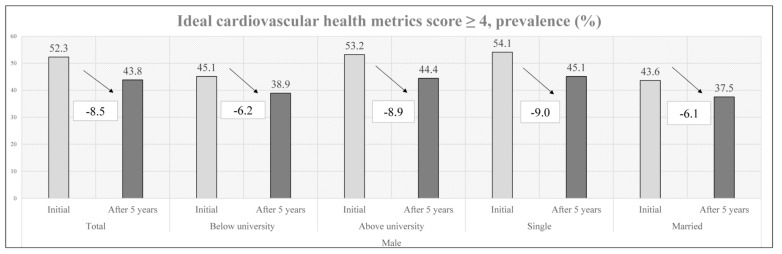
Early changes in males’ CVH metrics by education level and marital status.

**Figure 2:**
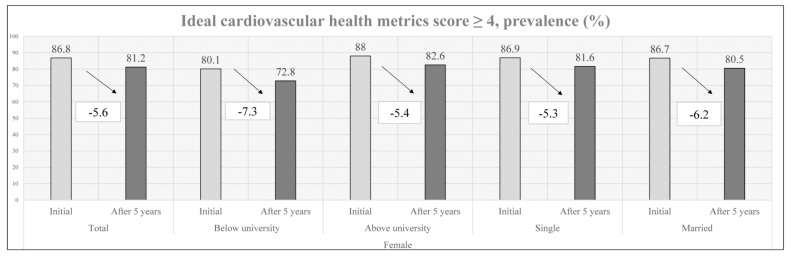
Early changes in female’ CVH metrics by education level and marital status.

In summary (Figure 1 and Figure 2), the initial prevalence of ideal CVH metrics was 52.3% and 86.8% and decreased to 43.8% and 81.2% after 5 years for males and females, respectively. In the subgroup analysis, males with less than a university education (6.2%) had a smaller ideal CVH metric decrease than males with more than a university education (8.9%), while females with more than a university education (5.4%) had a smaller ideal CVH metric decrease than females with less than a university education (7.3%). Married males (6.1%) had a smaller ideal CVH metric decrease than single males (9.1%), while single females (5.3%) had a smaller ideal CVH metric decrease than married females (6.2%). Moreover, some multivariable logistic regression models of unmarried/married status and less than a university education/more than a university education were shown in Table 5. The significant result was that the prevalence of an ideal CVH score ≥ 4 after 5 years was associated with participants with more than a university education. (Male: OR: 1.366, Female: OR: 1.763, *p* value < 0.001)

## 4. Discussion

This cohort study researched early changes in CVH metrics among young Asian adults and investigated the association between CVH metrics and sociodemographic factors (age, sex, marital status, and educational level). The main findings of our study were as follows: (1) the prevalence of ideal CVH metrics among young adults declined with age; (2) higher educational attainment and unmarried status were associated with a greater prevalence of ideal CVH metrics regardless of sex, but early CVH metric changes differed by sex, education level, and marital status.

Our first main result is in line with that of previous research focusing on early changes in CVH metrics among young adults [9,10]. In our study, the decrease in ideal CVH metrics prevalence among males was greater than that among females, similar to the findings of a Brazilian cohort study [10]. Sex is an essential factor affecting the prevalence of ideal CVH metrics and their changes. Females had a higher proportion of ideal CVH metrics than males in our study, in accordance with previous studies [31,32], which may be attributed to a higher prevalence of ideal smoking behaviors among women than among men. Therefore, cessation interventions should be focused on males. Moreover, women are more concerned about body shape and weight loss [33]. As a result, women tend to adopt better health habits and health-promoting behaviors more rapidly than men [34]. In the Asian population, the fact that younger females have a higher prevalence of CVH metrics is in accordance with Chinese studies, which showed that younger age and female sex were the two protective factors for CVH among adolescents and adults [35].

The prevalence of ideal CVH metrics among females was higher in all categories except for ideal PA when compared to their male counterparts. The National Health Interview Survey in Taiwan [36] found that young males spend more time on PA than young females. Some social and cultural factors influence women’s participation in PA in Taiwan [37]. Furthermore, after 5 years, ideal PA increased among females but decreased among males. Factors such as changes in equal education opportunities, family structure, and the government’s increasing focus on PA encouraged Asian women to increase their participation in PA [37]. Additionally, young adult men had less awareness of the PA recommendations, while women had greater adherence to PA [38].

The ideal diet is the metric most difficult to achieve [39]. The lowest prevalence of ideal PA and diet can also be seen in a Chinese cross-sectional study [16]. In our study, the low prevalence of an ideal diet among men and women is consistent with data showing poor dietary habits in the young adult population [9]. Furthermore, an ideal diet among females was more common than among males in our study, and an increasing prevalence of an ideal diet was observed after 5 years for both sexes. This result is consistent with that of a study that obtained data from the Nutrition and Health Survey in Taiwan (NAHSIT), which showed that both females and older individuals have higher adherence to healthy food guides [40].

In our study, ideal BMI, blood glucose, and cholesterol were more prevalent among females than males, similar to the results of a Korean study [41]. Men were generally less concerned about diet, weight, and body shape than women [42]. Moreover, BMI is often positively associated with blood glucose and cholesterol in the general population [43]. After 5 years, a decreasing prevalence of ideal BMI, blood glucose, and cholesterol was noted among both sexes. This is in line with previous studies showing an upward trend in the prevalence of fatness among young Taiwanese adults [44]. Young adult obesity is associated with cardiometabolic risk factors, such as diabetes mellitus and dyslipidemia [45]. The rise of obesity in early adulthood in Taiwan is attributable to a low level of PA and poor dietary habits, such as increased consumption of a Western diet [46]. In our study, we found a lower prevalence of baseline ideal BP among the male participants. This phenomenon may be due to sex differences in BP [47], and using the same cutoff value (120/80) would result in a higher prevalence of nonideal BP levels among males [10]. However, among the male participants, the increasing prevalence of ideal BP after 5 years was an interesting finding. We can only speculate as to why BP levels dropped among male participants in our study. It is worth mentioning that marital status and education were key factors in determining ideal BP changes in males.

Given that a large proportion of participants had more than a university education, they had a greater effect on the results of the five-year follow-up than participants with less than a university education, so their results were similar to those of all participants. Our study showed that ideal CVH scores were higher among the more educated participants than among their less educated counterparts. Inverse associations between low education levels and ideal CVH scores were also described in the National Health and Nutrition Examination Survey (NHANES) [48]. Moreover, in developing countries, college-educated participants had higher ideal CVH scores than participants with less than a high school education [49]. Additionally, our study revealed that education is a key factor that leads to a healthier lifestyle and that it has a greater impact on females than it does on males. Because women have fewer socioeconomic resources, such as power, authority, and income, education may improve women’s health more than men’s health [50].

Regarding detailed CVH metrics, the prevalence of an ideal diet increased after 5 years for both sexes for those with more than a university education. This result is consistent with that of a study showing that higher education was associated with higher adherence to daily healthy food guides [40]. The fact that education can encourage young adults to consume healthier foods is confirmed. Furthermore, participants who have higher levels of education may be from families with high socioeconomic status. Educated and wealthy parents can provide their children with more exercise opportunities and healthier food [8]. Therefore, highly educated participants may have had a healthier life while growing up, compatible with the results of the regression model.

Marriage is a life transition that may cause changes in diet, PA, and body weight [51]. Marital status is an important social characteristic for predicting health outcomes, including CVD. In several studies, being married was associated with lower odds of CVD than being single [52]. Our results agree with those of the British birth cohort study, which showed that marriage produces fewer health benefits for women than for men [53]. Married women are expected to perform housework and raise children, which can lead to poor health [54]. However, both spouses tend to be employed due to the financial needs of most modern households, resulting in a more equal balance of household chores and paid work between the spouses [55]. Due to trends in gender equality, recent research has reported that men and women have an equal impact on the health effects of marriage [56]. Furthermore, dissatisfaction with marriage and marriage quality has a significant impact on cardiovascular risk [57]. However, divorced and widowed participants were too few to include in our study population, and marital quality was not discussed in our study.

Marital status also influenced individual CVH metrics. In our study, marriage slowed the decrease in ideal PA among males and boosted the increase in ideal PA among females. These findings are in accordance with NHANES data, which revealed that married adults spent more time engaged in PA than single adults [58]. Being married was related to being aware of PA recommendations, but fewer young men than young women knew the PA recommendations [38]. Moreover, the ideal diet sharply increased among single study participants compared to married participants, regardless of sex. Young married adults may have too many financial and childcare responsibilities to maintain an ideal diet [59]. Moreover, this result was similar to that of education level; that is, singles were most closely aligned with the more highly educated group. Compared to less educated women, highly educated women tend to delay marriage [60]. One reason that highly educated people are apt to marry later is that they are waiting until after they are educated before they pursue marriage. Moreover, our findings are consistent with those of previous studies showing that married men were significantly more overweight than unmarried men. The marital role appears to influence obesity among men but not women [61]. Married men are less likely to perceive themselves as overweight, while women’s marital status is associated with weight-related desires and behaviors [62]. Therefore, married women with the strongest desire to lower their weight had the highest prevalence of ideal PA and ideal diet in our study. It is worth mentioning that marital status is a key factor in determining ideal BP changes in males. This is in line with a previous Chinese study that found that young single males seemed to have more ideal BP levels than their married counterparts [63]. Young married adults are likely to be overburdened with financial and childcare responsibilities, which may lead to interruptions in their education plans and have a negative impact on lifetime earnings and career development [59]. We confirm that chronic stress may lead to higher BP. Of note, in our study, ideal BP levels among males increased after 5 years regardless of education level, which was more pronounced among men with more than a university education. This could be because there were more single males in this group. Healthcare professionals may need to consider these social factors in addressing the risks for hypertension and cardiovascular disease prevention.Our study has some strengths. This is the first large-scale study to focus on early changes in CVH metrics and the cause of those changes in the young Asian adult population using validated questionnaires and laboratory evaluations. However, this study also has several limitations. First, health care is more available for our participants who have higher educational levels and net household income compared to the general Asian population. Therefore, our findings may not apply to the whole Asian population. Second, our participants were all of Han ethnicity. Due to ethnic differences in the prevalence of ideal CVH metrics, our findings may not apply to people of other ethnic groups [49,64]. Third, because PA was only measured by the transportation and leisure domains, PA was underestimated for some participants who engaged insubstantial PA while doing housework or at work. Fourth, our study did not include gradations of respondents’ socioeconomic status or education. Education level was distinguished by a university degree or above, and financial situation was divided by household income >1.2 million per year. Fifth, the presence of missing values is also a limitation of this study due to the retrospective study design. However, only complete CVH metric data (for all seven metrics) were available. There are no potential missing data on CVH metrics. Moreover, the missing ratios of education and marital status were both very small, 0.7 and 3.3, respectively, and can be ignored.

## 5. Conclusions

This is the first analysis of AHA CVH metrics in a large-scale young Asian adult population. Our study highlighted that age is inversely associated with CVH metrics in the young adult population. Higher educational attainment and unmarried status are associated with a greater prevalence of ideal CVH metrics regardless of sex, but early CVH changes differ by sex, education level, and marital status. The prevalence of CVH changes found early among young adults can be used to monitor CVH changes quickly. Furthermore, CVH metrics at young ages are a powerful indicator of CVD development in the adult population [8]. Therefore, effective programs promoting healthy lifestyles and health equality are needed to achieve optimal CVH metrics among young adults. Healthy lifestyles can focus on an ideal diet and ideal PA levels. Health equality can highlight strategies targeting CVH differently according to sex, education level, and marital status.

## Figures and Tables

**Table 1:** Demographic data with ideal cardiovascular health metrics among the participants.

	Male (*N* = 5042)	Female (*N*= 4958)	
**Numerical measurements, mean (S.D.)**			
Age	26.8 (2.4)	26.8 (2.4)	*p* = 0.542
BMI	23.5 (3.6)	20.5 (3.2)	*p* < 0.001
Total Cholesterol	181.6 (32.4)	176.9 (30.0)	*p* < 0.001
Glucose	95.9 (11.1)	91.1 (9.1)	*p* < 0.001
Systolic BP	120.4 (12.9)	107.3 (11.4)	*p* < 0.001
Diastolic BP	70.4 (9.3)	63.6 (8.4)	*p* < 0.001
**Categorical measurements, *N* (%)**			
Education^a^	4384 (86.9)	4188 (84.5)	*p* < 0.001
Finance^b^	355 (7.0)	335 (6.8)	*p* = 0.945
Marital status			*p* < 0.001
Single	4028 (79.9)	3381 (68.2)	
Married	850 (16.9)	1411 (28.5)	
Ideal CVH metrics			*p* < 0.001
0	55 (1.1)	2 (0.0)	
1	295 (5.9)	33 (0.7)	
2	728 (14.4)	123 (2.5)	
3	1327 (26.3)	496 (10.0)	
4	1621 (32.1)	1589 (32.0)	
5	913 (18.1)	2514 (50.7)	
6, 7	103 (2.0)	201 (4.1)	
Ideal PA	373 (7.4)	97 (2.0)	*p* < 0.001
Ideal BMI	3615 (71.7)	4569 (92.2)	*p* < 0.001
Ideal Diet	199 (3.9)	280 (5.6)	*p* < 0.001
Ideal Smoking	3294 (65.3)	4447 (89.7)	*p* < 0.001
Ideal Glucose	3717 (73.7)	4517 (91.1)	*p* < 0.001
Ideal Cholesterol	3746 (74.3)	3967 (80.0)	*p* < 0.001
Ideal BP	2458 (48.8)	4030 (81.3)	*p* < 0.001

Numerical variables are the means (S.Ds); categorical variables are *N* (%). CVH = cardiovascular health; PA: physical activity; BMI: body mass index; BP: blood pressure, a: education above university level, b: household income >1.2 million per year.

**Table 2:** Differences in ideal cardiovascular health metrics among the participants after 5 years.

	Male (*N* = 5042)			Female (*N*= 4958)		
	Initial	After 5 Years		Initial	After 5 Years	
Ideal CVH Metrics, *N* (%)			*p* < 0.001			*p* < 0.001
0	55 (1.1)	104 (2.1)		2 (0.0)	6 (0.1)	
1	295 (5.9)	495 (9.8)		33 (0.7)	63 (1.3)	
2	728 (14.4)	937 (18.6)		123 (2.5)	210 (4.2)	
3	1327 (26.3)	1296 (25.7)		496 (10.0)	655 (13.2)	
4	1621 (32.1)	1409 (27.9)		1589 (32.0)	1668 (33.6)	
5	913 (18.1)	720 (14.3)		2514 (50.7)	2108 (42.5)	
6,7	103 (2.0)	81 (1.6)		201 (4.1)	248 (5.0)	
Ideal PA	373 (7.4)	215 (4.3)	*p* < 0.001	97 (2.0)	156 (3.1)	*p* < 0.001
Ideal BMI	3615 (71.7)	3118 (61.8)	*p* < 0.001	4569 (92.2)	4331 (87.4)	*p* < 0.001
Ideal Diet	199 (3.9)	308 (6.1)	*p* < 0.001	280 (5.6)	379 (7.6)	*p* < 0.001
Ideal Smoking	3294 (65.3)	3305 (65.5)	*p* = 0.818	4447 (89.7)	4492 (90.6)	*p* = 0.129
Ideal Glucose	3717 (73.7)	3176 (63.0)	*p* < 0.001	4517 (91.1)	4182 (84.3)	*p* < 0.001
Ideal Cholesterol	3746 (74.3)	3168 (62.8)	*p* < 0.001	3967 (80.0)	3628 (73.2)	*p* < 0.001
Ideal BP	2458 (48.8)	2692 (53.4)	*p* <0.001	4030 (81.3)	3988 (80.4)	*p* = 0.284

Categorical variables are *N* (%). PA: physical activity; BMI: body mass index; BP: blood pressure; CVH = cardiovascular health.

**Table 3:** Differences in ideal cardiovascular health metrics among the participants divided by education level after 5 years.

		Male						Female					
		Less than a University Education (*N* = 627)			More than a University Education (*N* = 4384)			Less than a University Education (*N* = 735)			More than a University Education (*N* = 4188)		
		Initial	After 5 Years	*p*	Initial	After 5 Years	*p*	Initial	After 5 Years	*p*	Initial	After 5 Years	*p*
Ideal CVH metrics				0.021			<0.001			0.009			<0.001
CVH score	0	13 (2.1)	20 (3.2)		42 (1.0)	84 (1.9)		1 (0.1)	5 (0.7)		1 (0.0)	1 (0.0)	
	1	49 (7.8)	71 (11.3)		246 (5.6)	421 (9.6)		8 (1.1)	19 (2.6)		25 (0.6)	44 (1.1)	
	2	105 (16.7)	134 (21.4)		619 (14.1)	800 (18.2)		27 (3.7)	46 (6.3)		94 (2.2)	162 (3.9)	
	3	177 (28.2)	158 (25.2)		1144 (26.1)	1132 (25.8)		110 (15.0)	130 (17.7)		382 (9.1)	520 (12.4)	
	4	196 (31.3)	181 (28.9)		1415 (32.3)	1220 (27.8)		276 (37.6)	238 (32.4)		1301 (31.1)	1419 (33.9)	
	5	79 (12.6)	59 (9.4)		823 (18.8)	650 (14.8)		287 (39.0)	271 (36.9)		2211 (52.8)	1821 (43.5)	
	6,7	8 (1.3)	4 (0.6)		95 (2.2)	77 (1.8)		26 (3.5)	26 (3.5)		174 (4.2)	221 (5.3)	
Ideal PA		40 (6.4)	36 (5.7)	0.636	330 (7.5)	176 (4.0)	<0.001	15 (2.0)	34 (4.6)	0.006	82 (2.0)	120 (2.9)	0.007
Ideal BMI		471 (75.1)	406 (64.8)	0.001	3119 (71.1)	2688 (61.3)	<0.001	651 (88.6)	618 (84.1)	0.012	3890 (92.9)	3684 (88.0)	<0.001
Ideal Diet		21 (3.3)	28 (4.5)	0.308	177 (4.0)	278 (6.3)	<0.001	44 (6.0)	44 (6.0)	1.000	235 (5.6)	333 (8.0)	<0.001
Ideal Smoking		246 (39.2)	255 (40.7)	0.604	3021 (68.9)	3028 (69.1)	0.872	541 (73.6)	558 (75.9)	0.307	3875 (92.5)	3903 (93.2)	0.235
Ideal Glucose		458 (73.0)	385 (61.4)	<0.001	3237 (73.8)	2767 (63.1)	<0.001	641 (87.2)	580 (78.9)	<0.001	3845 (91.8)	3571 (85.3)	<0.001
Ideal Chol		463 (73.8)	404 (64.4)	<0.001	3257 (74.3)	2743 (62.6)	<0.001	603 (82.0)	546 (74.3)	<0.001	3339 (79.7)	3059 (73.0)	<0.001
Ideal BP		318 (50.7)	342 (54.5)	0.175	2123 (48.4)	2332 (53.2)	<0.001	592 (80.5)	585 (79.6)	0.648	3404 (81.3)	3372 (80.5)	0.374

Categorical variables are *N* (%). CVH = cardiovascular health; PA: physical activity; BMI: body mass index; Chol: Cholesterol; BP: blood pressure.

**Table 4:** Differences in ideal cardiovascular health metrics among the participants divided by marital status after 5 years.

		Male						Female					
		Single (*N* = 4028)			Married (*N* = 850)			Single (*N* = 3381)			Married (*N* = 1411)		
		Initial	After 5 Years	*p*	Initial	After 5 Years	*p*	Initial	After 5 Years	*p*	Initial	After 5 Years	*p*
Ideal CVH metrics				<0.001			0.001			<0.001			<0.001
CVH score	0	28 (0.7)	72 (1.8)		24 (2.8)	26 (3.1)		1 (0.0)	3 (0.1)		1 (0.1)	3 (0.2)	
	1	223 (5.5)	359 (8.9)		64 (7.5)	121 (14.2)		25 (0.7)	49 (1.4)		7 (0.5)	13 (0.9)	
	2	543 (13.5)	740 (18.4)		164 (19.3)	172 (20.2)		87 (2.6)	145 (4.3)		32 (2.3)	59 (4.2)	
	3	1054 (26.2)	1042 (25.9)		227 (26.7)	212 (24.9)		329 (9.7)	425 (12.6)		148 (10.5)	200 (14.2)	
	4	1329 (33.0)	1147 (28.5)		239 (28.1)	208 (24.5)		1061 (31.4)	1174 (34.7)		471 (33.4)	433 (30.7)	
	5	760 (18.9)	600 (14.9)		120 (14.1)	98 (11.5)		1748 (51.7)	1428 (42.2)		685 (48.5)	621 (44.0)	
	6,7	91 (2.3)	68 (1.7)		12 (1.4)	13 (1.5)		130 (3.8)	157 (4.6)		67 (4.7)	82 (5.8)	
Ideal PA		311 (7.7)	160 (4.0)	<0.001	50 (5.9)	45 (5.3)	0.598	70 (2.1)	94 (2.8)	0.058	25 (1.8)	56 (4.0)	<0.001
Ideal BMI		2973 (73.8)	2531 (62.8)	<0.001	526 (61.9)	483 (56.8)	0.034	3131 (92.6)	2955 (87.4)	<0.001	1288 (91.3)	1235 (87.5)	0.001
Ideal Diet		156 (3.9)	245 (6.1)	<0.001	37 (4.4)	54 (6.4)	0.067	166 (4.9)	250 (7.4)	<0.001	108 (7.7)	120 (8.5)	0.407
Ideal Smoking		2696 (66.9)	2707 (67.2)	0.794	486 (57.2)	497 (58.5)	0.589	3034 (89.7)	3059 (90.5)	0.309	1271 (90.1)	1291 (91.5)	0.193
Ideal Glucose		2997 (74.4)	2573 (63.9)	<0.001	596 (70.1)	495 (58.2)	<0.001	3110 (92.0)	2878 (85.1)	<0.001	1255 (88.9)	1157 (82.0)	<0.001
Ideal Chol		3048 (75.7)	2563 (63.6)	<0.001	577 (67.9)	503 (59.2)	<0.001	2701 (79.9)	2457 (72.7)	<0.001	1138 (80.7)	1061 (75.2)	<0.001
Ideal BP		1955 (48.5)	2185 (54.2)	<0.001	429 (50.5)	424 (49.9)	0.808	2742 (81.1)	2703 (79.9)	0.231	1145 (81.1)	1144 (81.1)	0.962

Categorical variables are *N* (%). CVH = cardiovascular health; PA: physical activity; BMI: body mass index; Chol: Cholesterol; BP: blood pressure.

**Table 5:** Logistic regression models of educational level and marital status.

		Predictors	B	S.E.	Wald	dF	*p* Value	Odds Ratio
Male	Education level ^a^	CVH score ≥4 ^c^	0.312	0.089	12.341	1	<0.001	1.366
		Age	0.168	0.017	96.620	1	<0.001	1.183
	Marital status ^b^	CVH score ≥4 ^c^	−0.132	0.083	2.537	1	0.111	0.876
		Age	0.517	0.025	444.421	1	<0.001	1.677
Female	Education level ^a^	CVH score ≥4 ^c^	0.567	0.093	37.592	1	<0.001	1.763
		Age	−0.045	0.017	7.096	1	0.008	0.956
	Marital status ^b^	CVH score ≥4 ^c^	0.014	0.087	0.024	1	0.877	1.014
		Age	0.438	0.018	588.483	1	<0.001	1.549

CVH = cardiovascular health; ^a^: Less than a university education/More than a university education; ^b^: Unmarried/married; ^c^: the prevalence of CVH score ≥ 4 after 5 years.

## Data Availability

All the data were collected from the MJ Health Management Institution between 2000 and 2016 (http://www.mjhrf.org/main/page/resource/en/#resource08 (accessed on 26 January 2023)).

## Supplementary Materials

The following supporting information can be downloaded at https://www.mdpi.com/article/10.3390/nu15030645/s1: Table S1: Five healthy dietary components reported by participants at baseline; Table S2: Logistic regression models of educational level and marital status.

## Abbreviations

CVDs: cardiovascular diseases; AHA, American Heart Association; CVH, cardiovascular health; PA, physical activity; BMI, body mass index; BP, blood pressure; NHANES, National Health and Nutrition Examination Survey
