# Supplementary material for: Prevalence of Ideal Cardiovascular Health Metrics among Young Asian Adults over 5 Years of Follow-Up
Source: Nutrients. 2023 Jan 27;15(3):645. doi: 10.3390/nu15030645 (PMC9920953; doi:10.3390/nu15030645)
Supplement: Supplementary file 1 [file nutrients-15-00645-s001.zip › nutrients-2107751-supplementary.pdf]

Supplementary Table S1: Five healthy dietary components reported by participants at baseline

	Male (n = 5,042)	Female (n= 4,958)	P value
Fruits and vegetables (≥ 450 g/d)	1470 (29.2)	1490 (30.1)	0.335
Fiber-rich whole grains (≥ 85 g/d)	119 (2.4)	59 (1.2)	<0.001
Sodium (< 1500 mg/d)	4426 (87.8)	4584 (92.5)	<0.001
Sugar-sweetened beverages (≤ 1 liter/wk)	2106 (41.8)	2625 (52.9)	<0.001
Fish (≥ 198 g/wk)	1194 (23.7)	1159 (23.4)	0.724

Categorical variables are N (%)
